# Myostatin inhibition therapy for insulin-deficient type 1 diabetes

**DOI:** 10.1038/srep32495

**Published:** 2016-09-01

**Authors:** Samantha K. Coleman, Irena A. Rebalka, Donna M. D’Souza, Namita Deodhare, Eric M. Desjardins, Thomas J. Hawke

**Affiliations:** 1 McMaster University, Department of Pathology and Molecular Medicine, Hamilton, ON, L8S 4L8, Canada

## Abstract

While Type 1 Diabetes Mellitus (T1DM) is characterized by hypoinsulinemia and hyperglycemia, persons with T1DM also develop insulin resistance. Recent studies have demonstrated that insulin resistance in T1DM is a primary mediator of the micro and macrovascular complications that invariably develop in this chronic disease. Myostatin acts to attenuate muscle growth and has been demonstrated to be elevated in streptozotocin-induced diabetic models. We hypothesized that a reduction in mRNA expression of myostatin within a genetic T1DM mouse model would improve skeletal muscle health, resulting in a larger, more insulin sensitive muscle mass. To that end, Akita diabetic mice were crossed with Myostatin^Ln/Ln^ mice to ultimately generate a novel mouse line. Our data support the hypothesis that decreased skeletal muscle expression of myostatin mRNA prevented the loss of muscle mass observed in T1DM. Furthermore, reductions in myostatin mRNA increased Glut1 and Glut4 protein expression and glucose uptake in response to an insulin tolerance test (ITT). These positive changes lead to significant reductions in resting blood glucose levels as well as pronounced reductions in associated diabetic symptoms, even in the absence of exogenous insulin. Taken together, this study provides a foundation for considering myostatin inhibition as an adjuvant therapy in T1DM as a means to improve insulin sensitivity and blood glucose management.

Type One Diabetes Mellitus (T1DM) results from the autoimmune-mediated destruction of the pancreatic beta cells rendering the individual hypoinsulinemic. In the absence of insulin, blood glucose levels rise dramatically as glucose cannot readily be taken up into the major insulin sensitive tissues: skeletal muscle, liver and adipose. Like other forms of diabetes, individuals with T1DM are at a significantly elevated risk for a number of comorbidities including nephropathy, neuropathy, cardiovascular disease, myopathy, retinopathy and non-healing dermal wounds^1^. Appropriate health care provision for T1DM patients is associated with significant costs to healthcare systems and the early onset of T1DM results in a longer exposure to endocrine and metabolic derangements, ultimately increasing disease burden. In fact, despite readily available insulin therapies, it is estimated that the number of life-years lost is still greater than 10 years^2^.

Although T1DM is primarily a disease of insulin deficiency, T1DM patients are insulin-resistant compared with non-diabetic subjects; with insulin resistance observed both during puberty and in those with long-standing T1DM^3^,^4^,^5^,^6^,^7^,^8^. While insulin resistance in T1DM was previously thought to be directly related to adiposity and elevated HbA1c^3^,^8^,^9^, recent evidence has demonstrated insulin resistance in multiple tissues of those with T1DM despite good glycemic control and a BMI similar to that of non-diabetics^10^,^11^. The exact mechanism(s) underlying insulin resistance in T1DM have yet to be fully elucidated. Regardless of the mechanism(s) instigating the reduced insulin sensitivity in T1DM^4^,^6^,^12^,^13^,^14^, there is robust data demonstrating that insulin-resistant persons with T1DM are at a significantly greater risk of developing microvascular and macrovascular complications^4^,^15^,^16^,^17^,^18^,^19^,^20^,^21^,^22^. Thus, strategies to improve insulin sensitivity in those with T1DM is of the utmost clinical significance.

Myostatin is a secreted growth and differentiating factor (GDF-8) belonging to the transforming growth factor-beta (TGF-β) superfamily. Predominantly expressed by skeletal muscle, myostatin is a negative regulator of muscle growth such that animals in which the myostatin gene is disrupted display significant increases in muscle mass^23^. Elevations in myostatin expression have been observed in a number of disease states associated with impaired muscle health including Type 2 and streptozotocin-induced diabetes^24^,^25^,^26^,^27^,^28^. Furthermore, myostatin inhibition in rodent models of lipodystrophy and obesity resulted in measurable improvements in insulin sensitivity and an upregulation of glucose transporters^29^,^30^,^31^,^32^.

We therefore hypothesized that T1DM mice exhibiting reduced myostatin mRNA expression would demonstrate improved skeletal muscle health resulting in increased insulin sensitivity and reduced blood glucose. The findings presented here support the hypothesis that reducing the expression of myostatin prevents the loss of skeletal muscle mass observed in T1DM, as well as significantly increases insulin sensitivity and glucose transporter density. These positive changes in T1DM skeletal muscle result in significant reductions in blood glucose levels and other diabetic symptoms (hyperphagia, polydipsia) despite the absence of exogenous insulin.

## Results

### Reducing Myostatin mRNA Results in Maintenance of Muscle Mass and Type IIB Fiber Content in Diabetic Mice

**Figure 1:**
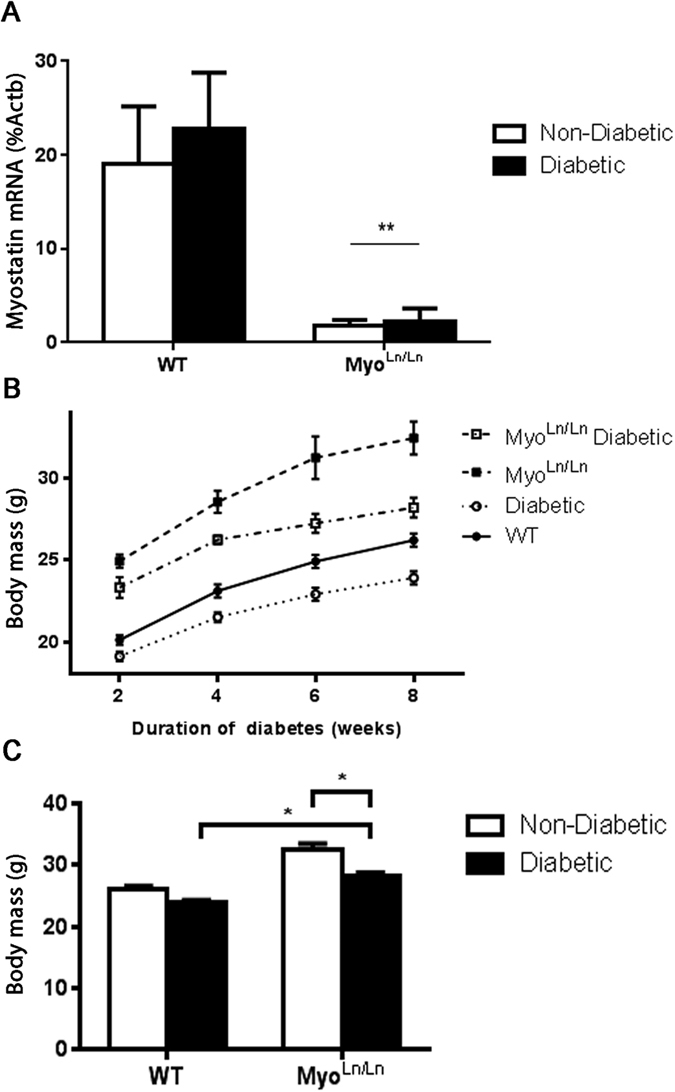
Myo^Ln/Ln^ animals demonstrate increases in body mass. Myo^Ln/Ln^ diabetic animals do not have the same decreases in body mass typically observed in diabetes. (**A**) qPCR of skeletal muscle myostatin mRNA expression compared to beta-actin. Two-way ANOVA, **indicates main effect of Myo^Ln/Ln^ genotype. p < 0.05; N = 4–7. (**B**) Body mass changes from 2 to 8 weeks of uncontrolled diabetes. N = 4–16. (**C**) Body mass at 8 weeks of diabetes. Two-way ANOVA with Bonferroni post-hoc test. *Indicates p < 0.05; N = 5–16.

Figure 1 demonstrates the successful reduction of myostatin mRNA and the resultant increases in body mass observed in our myostatin-reduced animals. Wilkes *et al.* have previously characterized and described the myostatin Ln/Ln model^33^. Briefly, an ENU-induced mutation results in abnormal splicing and thus an elongated mRNA product^33^. This elongated product has a premature stop codon, producing a truncated, non-functional protein^33^. In agreement with this previous work^33^, Myo^Ln/Ln^ mice exhibited a significant drop in *Myostatin* mRNA expression in the muscle (Fig. 1A). With respect to body mass, Myo^Ln/Ln^ mice demonstrated large gains in body mass over the 8 week experimental period compared to WT animals (Fig. 1B). This finding was also true when comparing the Myo^Ln/Ln^ diabetic animals to Akita diabetic mice without myostatin deficiency. After 8 weeks of uncontrolled diabetes, Myo^Ln/Ln^ diabetic animals had significantly larger body mass than WT diabetics (Fig. 1C).

**Figure 2:**
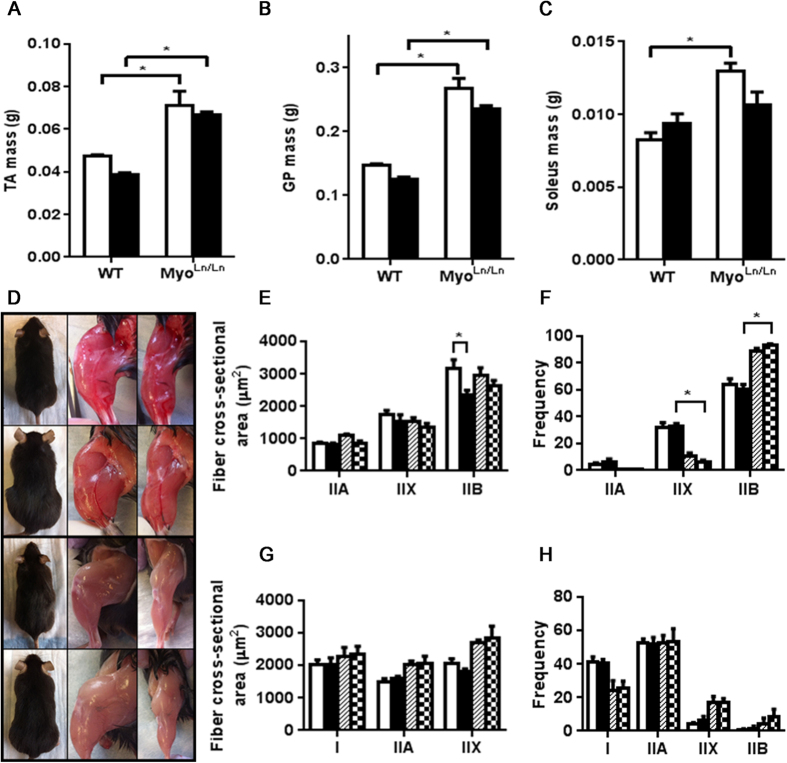
Muscles of Myo^Ln/Ln^ animals are much larger than those with normal myostatin expression. Diabetic Myo^Ln/Ln^ animals have significantly larger muscles than those of the Akita mouse. (**A**) TA mass at 12 weeks of age. N = 5–11.(**B**) GP mass at 12 weeks of age. N = 5–8. (**C**) Soleus mass at 12 weeks of age. N = 4–8. (**D**) Pictures of mouse cohorts. Top panels – WT. Second from top – Myo^Ln/Ln^. Second from bottom – Diabetic; bottom – Myo^Ln/Ln^ Diabetic. (**E**) TA cross-sectional area N = 3–4. White bar – WT, black bar – diabetic, hatched bar - Myo^Ln/Ln^, checkered – Myo^Ln/Ln^ diabetic. (**F**) TA fiber type proportion. N = 4. (**G**) Soleus cross-sectional area. Note: The number of IIB fibers was too few within the soleus to compare cross-sectional area (some sections had no IIB fibers). N = 3–4. (**H**) Soleus fiber type proportion. N = 3–4. For all graphs two-way ANOVA with Bonferroni post-hoc test was performed. *Indicates p < 0.05.

The masses of the TA, GP and soleus muscles all demonstrated a similar trend as the body mass of each respective cohort; confirming the muscle phenotype associated with myostatin deficiency (Fig. 2A–C). In the case of the Myo^Ln/Ln^ diabetic mice there were significant increases of the TA and GP muscle mass compared to Akita animals. As was hypothesized, the Myo^Ln/Ln^ background in diabetic mice resulted in an attenuation of muscle mass loss that is traditionally seen in diabetic mice^34^. Phenotypically, these mice and their muscles are visibly much larger in the myostatin deficient animals than in WTs (Fig. 2D).

We and others have previously noted that diabetic mice (including STZ-induced and Akita mouse models) exhibit a reduction in Type IIB (glycolytic) fiber areas and/or number^34^,^35^,^36^. In the present study, we observed the maintenance of IIB fiber type areas in Myo^Ln/Ln^ diabetics (vs. Akita; Fig. 2E,G) and, consistent with previous reports^37^,^38^,^39^, an increased percentage of IIB muscle fiber type compared to mice without the Myo^Ln/Ln^ background (Fig. 2F,H).

### Increased Insulin Sensitivity, Decreased Blood Glucose and Reduced Diabetic Symptoms in Diabetic Mice with Reduced Myostatin mRNA

**Figure 3:**
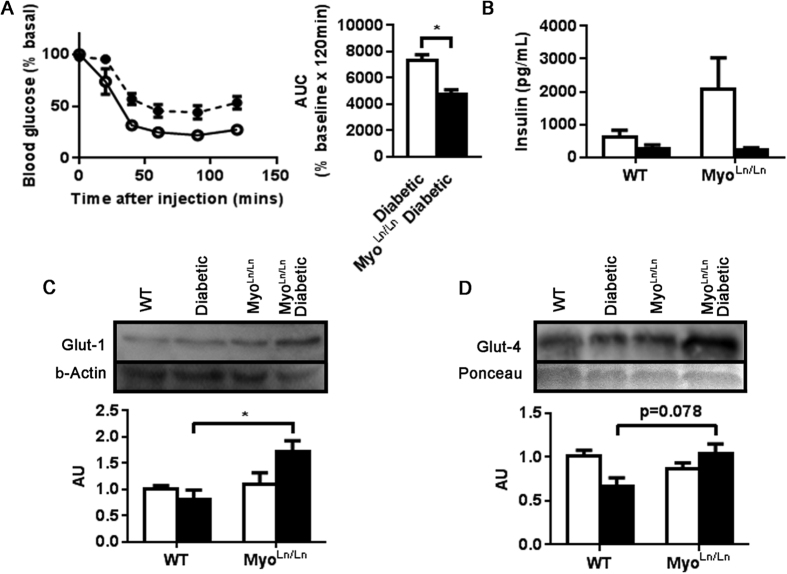
Myo^Ln/Ln^ diabetic animals have greater insulin sensitivity to bolus insulin injection and increased expression of key glucose transporters. (**A**) Blood glucose measures following insulin tolerance test. Broken line, black circle – diabetic; Unbroken line, white circle – Myo^Ln/Ln^ diabetic. AUC – area under the curve. N = 5–6. Unpaired t-test. *Indicates p < 0.05. (**B**) Plasma insulin measurement. N = 4. White bar – non-diabetic, black bar – diabetic (**C**) Glut-1 protein expression. N = 3–4. Two-way ANOVA with Bonferroni post-hoc test. *Indicates p < 0.05. (**D**) Glut-4 protein expression. N = 4.

In response to an intraperitoneal insulin tolerance test, Myo^Ln/Ln^ diabetic animals displayed a greater reduction in blood glucose than Akita diabetic mice (Fig. 3A). It is notable that Akita mice are not completely void of insulin; producing very low levels consistent with fasting in a WT mouse. There was no difference between diabetic groups (Fig. 3B) in circulating insulin levels. Compared to Akita mice, Myo^Ln/Ln^ diabetic mice also displayed an increased expression of key glucose transporters with regard to both insulin-independent (Glut-1; Fig. 3C) and insulin-dependent (Glut-4; Fig. 3D) glucose uptake.

**Figure 4:**
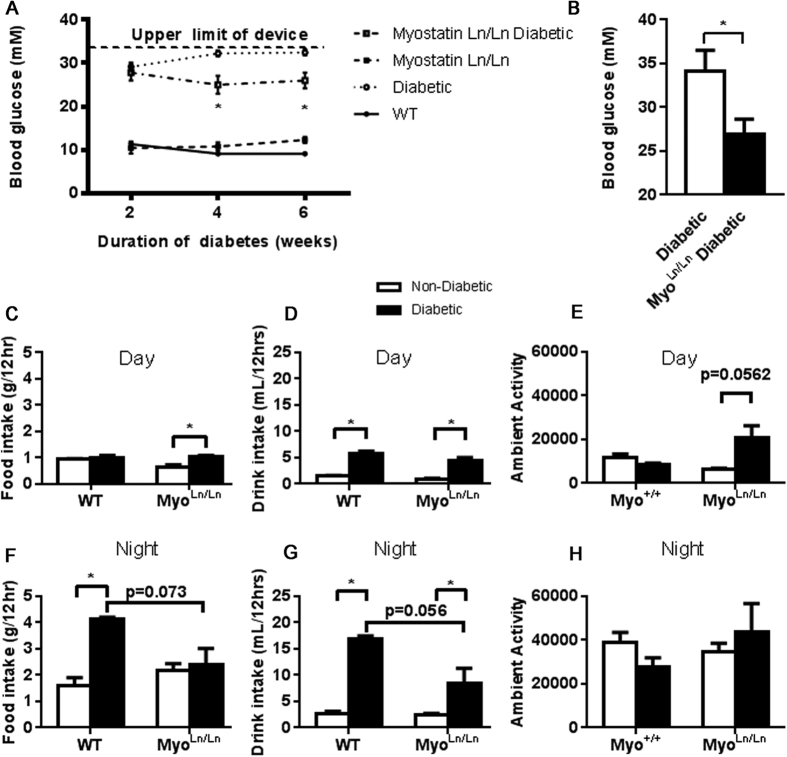
Myo^Ln/Ln^ diabetic mice have significantly reduced blood glucose levels and decreased food and drink consumption. (**A**) Blood glucose measures over a 4 week period. Note the device used for these measurements had an upper limit of 34 mM. Black circle – WT; white circle – diabetic; black square – Myo^Ln/Ln^; white square – Myo^Ln/Ln^ diabetic. N = 4–16. Two-way ANOVA test with Bonferroni post-hoc. *Indicates p < 0.05. (**B**) YSI measure of blood glucose at 8 weeks of diabetes. No upper limit. N = 3–4. (**C**) Daytime food intake. Non-diabetic – white bar, diabetic- black bar. N = 4. Two-way ANOVA test with Bonferroni post-hoc for all remaining graphs. *Indicates p < 0.05. **D.** Day-time drink intake, N = 4. (**E**) Day-time activity, N = 4. (**F**) Night-time food intake, N = 4. (**G**) Night-time drink intake, N = 4. (**H**) Night-time activity, N = 4.

Blood glucose measures in the Myo^Ln/Ln^ diabetic animals were significantly reduced compared to Akita mice beginning as early as 2 weeks following onset of diabetes and remained consistently and significantly reduced at all time-points recorded in this study (Fig. 4A). Since the glucometer used for blood glucose measures had an upper recording measurement limit of 34 mM and measured values in Akita mice were often greater than the measurable limit, a YSI analyzer was used on samples from 8 weeks of uncontrolled diabetes. The YSI analyzer confirmed the decreased blood glucose measures in the Myo^Ln/Ln^ diabetic animals compared to Akita diabetic animals without myostatin deficiency (Fig. 4B). Akita mice exhibit hyperphagia and polydipsia; characteristic of uncontrolled diabetes (Fig. 4C,D,F,G). Consistent with the significant decrease in blood glucose, Myo^Ln/Ln^ diabetic mice displayed a significant attenuation of hyperphagia and polydipsia (Fig. 4C,D,F,G) which was more clearly evident during the mouse’s active, night-time, period. These animals also demonstrated an increased level of activity during the day-time (inactive) period (Fig. 4E), although this was not significant during the mouse’s night-time (active) period (Fig. 4H).

### No Change in Circulating Leptin or White Adipose Tissue ‘Browning’

**Figure 5:**
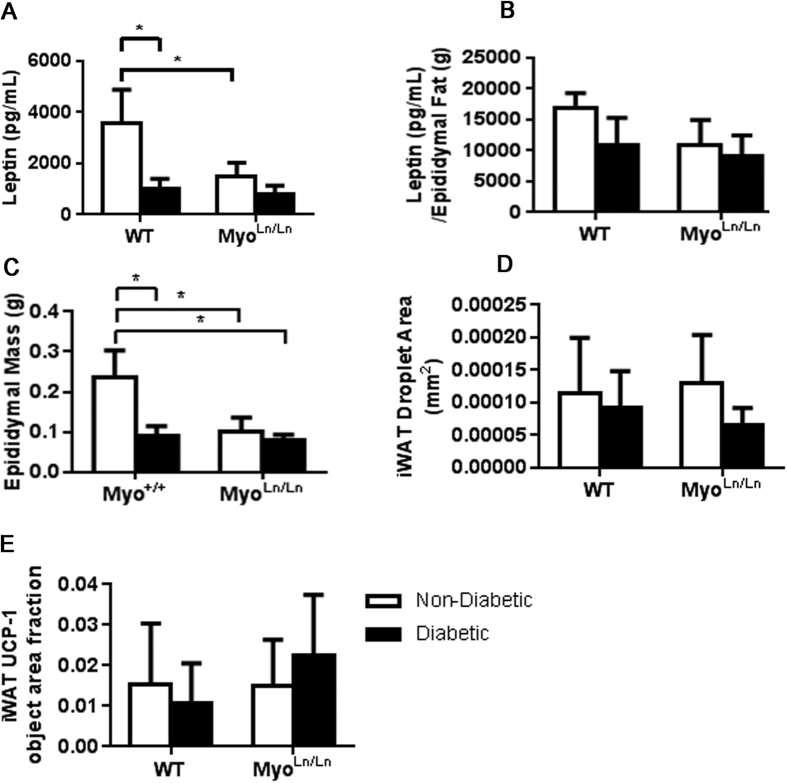
Despite lower glucose levels, plasma leptin concentration remains low and Myo^Ln/Ln^ diabetic mice do not show significant evidence of “beiging” iWAT stores. For all graphs two-way ANOVA with Bonferroni post-hoc test was performed. *Indicates p < 0.05. White bar – diabetic; black bar – non-diabetic. (**A**) Plasma leptin measures at 8 weeks of diabetes. N = 3–4. (**B**) Leptin measurement normalized to the mass of the epididymal fat mass N = 3–4. (**C**) Epididymal fat mass, N = 7–12. (**D**) Area of iWAT lipid droplets. N  = 4–6, male and female. (**E**) Object area fraction of UCP-1 stain in iWAT tissue. N = 4–8, male and female.

Though our hypothesis was that a larger, more insulin-sensitive muscle mass, was the mechanism responsible for the benefits observed, we also tested other possible mechanisms that could account for the beneficial outcomes noted in diabetic mice in the Myo^Ln/Ln^ background. A previous report from Unger’s research team^40^ demonstrated that restoring leptin levels in Non-Obese Diabetic (NOD) mice could alleviate diabetic symptoms by reducing circulating glucagon. We did not observe any significant differences in leptin values between our diabetic groups (Akita: 1016.91 pg/mL ± 193.33 pg/mL, Myo^Ln/Ln^ diabetic: 778.68 pg/mL ± 178.59 pg/mL; p = 0.4004; Fig. 5A) suggesting that altered leptin levels were not involved in the improvement in diabetic symptoms in Myo^Ln/Ln^ diabetic mice. When normalized to the mass of the epididymal fat, leptin values remained low in the animals, although not significantly different from any other group (Fig. 5B). Epididymal mass itself remains very low in the Myo^Ln/Ln^ diabetic animals, although interestingly not significantly different from diabetic animals.

Another possible mechanistic explanation for the beneficial effects of myostatin inhibition on circulating blood glucose was the ‘beiging’ of inguinal white adipose tissue (iWAT) stores^41^,^42^. No difference between groups was noted in lipid droplet sizes (Fig. 5D) or UCP-1 protein expression (Fig. 5E) in the iWAT depots, suggesting that a beiging of iWAT was not playing a primary role in reducing diabetic symptoms.

## Discussion

Despite the significance of a healthy, insulin-sensitive, skeletal muscle mass in the management of circulating blood glucose, improving the health of our largest metabolic organ is often overlooked as a therapeutic target in the management of T1DM. This study demonstrates a novel means for attenuating the diabetic phenotype through improvements in muscle health via reductions in myostatin mRNA expression. By decreasing the mRNA expression of myostatin in the skeletal muscle of T1DM mice, the loss of skeletal muscle and whole body mass was attenuated in comparison to diabetic Akita mice. Furthermore, Myo^Ln/Ln^ diabetic animals demonstrated improved insulin sensitivity with concomitant increases in skeletal muscle Glut1 and 4 glucose transporters. The maintenance of muscle mass, along with the changes in glucose handling abilities, in Myo^Ln/Ln^ diabetic mice resulted in a decline in diabetic symptoms, including reductions in blood glucose levels and a decrease in hyperphagia and polydipsia. It is of interest to note that although elevated myostatin expression has been reported in streptozotocin-induced T1DM^25^,^43^,^44^, we did not observe an increase in skeletal muscle myostatin mRNA expression. Our findings indicate that the positive outcomes reported herein are not dependent on the repression of elevated myostatin levels, but rather a reduction of myostatin mRNA to sub-normal levels (regardless of the starting level of myostatin mRNA). These findings open up considerable potential for investigating the pharmacological reduction of myostatin in T1DM as an adjuvant therapy that enhances insulin sensitivity and reduces the requirements of exogenous insulin in T1DM persons.

While insulin resistance is considered a defining trait of those with T2DM, individuals with T1DM also develop insulin resistance and this complication is now established as a primary contributor to the development of micro- and macrovascular complications^4^,^15^,^16^,^17^,^18^,^19^,^20^,^21^,^22^. In fact, an association between insulin sensitivity and microvascular complications has been known for some time as Martin *et al.*^22^ showed that microvascular complications were associated with insulin resistance in long-standing T1DM. With respect to macrovascular complications, T1DM is associated with a 10-fold increase in cardiovascular disease (CVD) compared to non-diabetics^45^,^46^ and though an association between hyperglycemia and cardiovascular disease has been suggested^47^, controlled clinical trials of T1DM and T2DM have not demonstrated a reduction in CVD occurrence with long-term intensive diabetes therapy, suggesting that other factors play a critical role in complications progression^10^,^48^. A longitudinal cohort study of T1DM adults (CACTI study) reported that T1DM patients are insulin-resistant (a finding that was independent of their current glycemic control), and the degree of insulin resistance predicted the extent of coronary artery calcification^10^,^48^. The findings of the CACTI study support data from the EDC and DCCT trials, which also demonstrated strong associations between insulin sensitivity and coronary artery disease in adults with T1DM^5^,^20^. These findings in adults with T1DM are also consistent with the recent work of Specht *et al.*^21^, who found that higher estimated insulin sensitivity in adolescents with T1DM is inversely associated with CVD risk factors. With the relationship between complications and insulin resistance established, potential adjuvant strategies which would improve the insulin sensitivity of those with T1DM would be considered of paramount importance. The data presented here demonstrating that reductions in skeletal muscle myostatin mRNA expression improve insulin sensitivity and increased glucose uptake in T1DM mice support this initiative.

The use of pharmacological myostatin inhibition as a therapeutic strategy for T1DM is often stigmatized by the belief that is would come with an unwanted hypermuscular phenotype. It should be noted that clinical trials of myostatin-based therapies do not appear to have the same dramatic effect as is observed in mice, dogs and cattle with complete myostatin ablation. For example, BYM338 trial patients displayed only a small (~6%) increase in their thigh muscle volume, but measurable improvements in walk time and distance^49^. Thus, the improved functional outcomes were noted without unabated muscle growth and were achieved with only bi-monthly injections^49^.

Wang *et al.*^28^ recently demonstrated that although myostatin pathway inhibition (Activin receptor type IIb antagonist) was capable of restoring muscle size in STZ-treated mice, it did not improve hyperglycemia. The lack of effect of myostatin inhibition on hyperglycemia in their study is in contrast to the current findings, and previous work using mouse models of obesity and lipodystrophy^29^,^30^,^31^,^32^,^33^ (including studies authored by the same group [31, 32]). The exact reasons for the differential results are still unclear but may be related to the elevated glucocorticoids reported in their STZ-induced diabetes model^28^. In terms of type II diabetes, myostatin inhibition has been seen to prevent obesity and hyperglycemia^30^,^31^,^32^,^33^. This has been hypothesized to be due to irisin-mediated pathways^42^. While we investigated this pathway to determine if irisin was playing a role in these animals, no observable differences were seen (data not shown).

Previous reports from Unger and others have highlighted dramatic improvements in diabetic symptoms (severe hyperglycemia and ketosis) with exogenous leptin administration to T1DM mice^50^. Consistent with Unger’s group^50^, we also observed a significant decrease in circulating leptin levels in our Akita diabetic mice compared to WT. However, both Myo^Ln/Ln^ and Myo^Ln/Ln^ diabetic mice display significantly less plasma leptin than WT mice. In fact, leptin levels in mice with the Myo^Ln/Ln^ background were not significantly different from Akita mice suggesting that a restoration of leptin levels was not the mechanism accounting for the attenuation of diabetic symptoms noted in the present study.

While the Akita mouse model is characterized by the development of T1DM at approximately 4 weeks of age due to misfolding and endoplasmic reticulum accumulation of insulin, these mice are still able to produce low levels of insulin. Plasma insulin levels in the Myo^Ln/Ln^ diabetic were not different from that of diabetic controls, however, insulin sensitivity was elevated in the Myo^Ln/Ln^ diabetics following bolus injection of insulin. Consistent with this observation, Myo^Ln/Ln^ diabetic mice also exhibited increased expression of key glucose transporters Glut-1 and Glut-4 for both insulin-independent and -dependent methods of glucose transport, respectively. Thus, the mice were more able to adapt to glycemic fluctuations. While the exact mechanism of this result is unclear, this has previously been seen in animals with reduced myostatin^29^. If paired with exogenous insulin as an adjuvant therapy, prospective myostatin inhibition therapies should improve insulin sensitivity and ultimately aid in glucose control. Together, this would significantly delay (or maybe even eliminate) the onset of insulin resistance and the associated complications seen in T1DM patients^4^,^15^,^16^,^17^,^18^,^19^,^20^,^21^,^22^.

In recent years, the pharmaceutical industry has focused a tremendous amount of their interest in the repurposing of existing compounds to decrease development costs and speed the time to clinic^51^. Our proposed research builds the foundation for the use of myostatin inhibitors as an adjuvant therapy to reduce the development and progression of diabetic complications through reductions in blood glucose and improvements of insulin sensitivity, thus enhancing the lifespan of those suffering from T1DM.

## Methods

### Animal Care

Male C57BL/6J-Mstn^lean^/J mice (hereafter referred to as Myo^Ln/Ln^ mice) were bred with C57BL/6-Ins2^Akita/+^/J mice (hereafter referred to as Akita mice), both purchased from the Jackson Laboratories (Bar Harbor, ME). Myo^Ln/Ln^ mice have a chemically induced mutation in the myostatin gene which results in an improperly spliced, truncated, loss-of-function protein, as characterized previously^33^. Akita mice spontaneously develop T1DM at approximately four weeks of age (due to a mutation in the *insulin 2* gene). Four groups of male progeny from the cross were analyzed: Myo^Ln/Ln^, Myo^Ln/Ln^ diabetic, Akita (diabetic) and wildtype (WT) mice. Animals were maintained on 12 hr light/dark cycle at 21°C with access to food and water ad libitum. Body mass and blood glucose were measured every two weeks beginning at approximately six weeks of age (two weeks of uncontrolled diabetes) until approximately twelve weeks of age (eight weeks of uncontrolled diabetes). Blood glucose was measured via tail-nick (OneTouch Ultra glucometer; maximum 34 mM; Johnson & Johnson). Animals were not fasted; however, blood plasma samples were collected at 9:30am every two weeks, concurrent with the time points for previously mentioned body mass and blood glucose measures. Blood plasma samples from 12 weeks of age were analyzed using the YSI glucose monitor (YSI Life Sciences, Yellow Springs, Ohio) for more accurate blood glucose readings with higher upper limit. All experiments performed were approved by the McMaster University Animal Care Committee in accordance with the Canadian Council for Animal Care Guidelines.

### Food/Water and Activity Measurements

At approximately 8–12 weeks of age, animals were placed within the comprehensive laboratory animal monitoring system (CLAMS) cages. Animals were acclimatized to the cages overnight before being maintained in these cages for three days while food intake and drink intake were recorded at twenty minute intervals while ambient activity (infrared beam breaks) was recorded throughout the experimental period.

### Insulin Sensitivity

Mice underwent an intraperitoneal insulin tolerance test at four weeks of uncontrolled diabetes (8 weeks of age). Mice were fasted for six hours prior to the test and blood glucose was measured via tail nick (as described above) before the mice were injected with 0.5 units/kg insulin (NovoRapid; Novo Nordisk) to record baseline levels. Blood glucose was measured 20, 40, 60, 90 and 120 minutes following injection.

### Tissue Collection

At 12 weeks of age, animals were euthanized via cervical dislocation and blood was collected. Tibialis anterior (TA), gastrocnemius plantaris (GP) and soleus muscles were harvested, weighed and then coated in optimal cutting temperature compound and frozen in liquid nitrogen cooled 2-methylbutane. Quadriceps muscles were harvested and flash frozen in liquid nitrogen. Inguinal white adipose tissue (iWAT) was fixed in 4% PFA and embedded in paraffin.

### Histology and Immunofluorescence

Muscle fiber type (as determined by myosin heavy chain (MyH) staining) proportion and cross-sectional area was determined in TA and soleus muscles. 10 μm thick sections were obtained. Slides were blocked for 1 hr in 10% normal goat serum (NGS) (Vector Laboratories; Burlingame, California) before application of primary antibody cocktail for Myosin Heavy Chain I, Myosin Heavy Chain IIa, and Myosin Heavy Chain IIb (BA-F8, SC-7A, BF-F3 respectively; Developmental Studies Hybridoma Bank, University of Iowa) for two hours at room temperature (RT). Slides were washed in phosphate buffered saline before the application of a secondary antibody cocktail Alexa 350 IgG2b, Alexa 488 IgG1, Alexa 594 IgM (A21140, A21121, A21044 respectively; Molecular Probes by Life Technologies) for one hour at RT. Slides underwent a final wash before being mounted.

iWAT samples were sectioned at 10 μm thickness and stained with hematoxylin and eosin. Separate iWAT slides were further stained for UCP-1. Slides were prepared similar to that of above, however with primary antibody for UCP-1 (ab155117) and secondary antibody Alexa 488 (A-11008). Slides were counterstained with DAPI before being mounted.

### Insulin ELISA

Blood plasma samples from all animals at 12 weeks of age were analyzed for plasma insulin levels using an insulin ELISA kit (Crystal Chem Inc. Ultra Sensitive Mouse Insulin ELISA Kit).

### Milliplex Map Kit

Blood plasma samples from all animals at 12 weeks of age (8 weeks of diabetes) were analyzed for leptin levels using the Milliplex Metabolic Magnetic Bead Panel (MMHMAG-44 K, Millipore; Billerica, MA.).

### Image Analysis

All images were captured using the Nikon 90i-eclipse microscope (Nikon Inc.; Melville, NY) and analyzed with the NIS Elements software (Nikon Inc.). Negative controls (lack of primary antibody), which demonstrated lack of signal, were used to create a standard to set threshold settings. Fibers were manually circled using the NIS Elements software. iWAT adipose droplet size was quantified using ImageJ software (1.48 v).

### Western Blots

Flash-frozen muscle samples from the quadriceps muscle were crushed, dissolved in RIPA buffer (R0278-50ML Sigma Aldrich; St. Louis, MO) with phosphatase inhibitor cocktail tablet (PhosSTOP EASYpack Roche; Basel, Switzerland) and protease inhibitor cocktail tablet (cOmplete MINI Roche). Samples were sonicated and assessed for protein content using Bradford Assay. 20 μg of tissue was loaded, resolved by SDS-PAGE, transferred to PVDF membrane and probed with antibodies for GLUT4 (07-1404, Millipore; Billerica MA) and GLUT1 (ab652, Abcam Inc.).

### mRNA

Quantitative real-time PCR (qPCR) was used as previously described^52^ with the following Taqman primers obtained from Invitrogen: Mm01254559_m1 (Mstn) and Mm00607939_s1 (Actb). Relative gene expression was calculated using the comparative CT (*ddCt*) method, where values were normalized and expressed as a percentage of the housekeeping gene (Actb).

### Statistics

All statistical tests were performed using GraphPad Prism 6.05. Student t-test (unpaired) was used in the case of one variable comparisons, two-way ANOVA was performed with Bonferroni post-hoc analysis in the case of two variable comparisons. P < 0.05.

## Additional Information

**How to cite this article**: Coleman, S. K. *et al.* Myostatin inhibition therapy for insulin-deficient type 1 diabetes. *Sci. Rep.* **6**, 32495; doi: 10.1038/srep32495 (2016).
